# Flap of the sternocephalicus muscle in the repair of a partial defect in the trachea of a rabbit (*Oryctolagus cuniculus*)

**DOI:** 10.1590/acb390324

**Published:** 2024-02-05

**Authors:** Gustavo Fernandes, Ariadne Rein, Gabriel Luiz Montanhim, Marcelo Carrijo da Costa, Marcella Dall’Agnol Leite, Nicolle Pereira Soares, Paola Castro Moraes

**Affiliations:** 1 Universidade Estadual Paulista “Júlio de Mesquita Filho” – Faculty of Agricultural and Veterinary Sciences – Department of Veterinary Clinic and Surgery – Jaboticabal (SP), Brazil.; 2 Universidade Federal de Uberlândia – College of Veterinary Sciences – Department of Veterinary Pathology – Uberlândia (MG), Brazil.

**Keywords:** Endoscopy, General Surgery, Respiratory System, Surgical Flaps

## Abstract

**Purpose:**

The current study aimed at evaluating the repair of a partial defect of the trachea with a muscle flap, an advanced technique that employs combined suture patterns.

**Methods:**

Sixteen healthy male New Zealand white rabbits were used as an experimental model. A partial defect in the trachea within the ventral region of the fourth to eighth tracheal ring was created. Subsequently, repair was initiated with a flap of the sternocephalicus muscle. The animals were divided into four groups for postoperative evaluation using clinical, tracheoscopic, and histopathological analyses. Each group was separated according to the time of euthanasia, programmed at interval of seven (G7), 15 (G15), 30 (G30), and 60 days (G60).

**Results:**

One animal from the G60 group died, whereas the other animals had good surgical recovery without serious changes in the breathing pattern. The major clinical signs observed were stridor and coughing. Tracheoscopy revealed secretions in the tracheal lumen, exuberant granulation, and stenosis. Histopathological analysis showed growth of the ciliary respiratory epithelium at the flap site 30 days after implantation.

**Conclusions:**

Partial repair showed satisfactory results owing to the anatomical location of the muscle, adequate vascular support, and structural and physiological maintenance without serious changes in the respiratory system.

## Introduction

The main causes of tracheal injuries are bites, car accidents, injuries from collars and neoplasms, and iatrogenic factors associated with anesthetic complications such as intubation or surgical procedures in the cervical region^1^^,^ 2. Tracheal injuries can be potentially fatal, requiring quick care, surgical approaches, and fundamental actions for the patient’s survival^3^.

Several surgical alternatives are used in tracheal repair, either through sutures or resection followed by anastomosis. These methods are employed in addition to autogenous skin, muscle, and cartilage grafts^4^. One of the muscles that are widely used in the reconstruction of the larynx and trachea is the sternohyoid. However, careful dissection is necessary to preserve its blood supply, which, if compromised, can cause ischemic atrophy and local fibrosis^5^^,^ 6.

The use of complementary examinations such as endoscopy provides a new method of internal evaluation of cavities and hollow organs, with a minimally invasive and non-traumatic diagnostic and therapeutic purpose^7^. Through tracheoscopy, it is possible to diagnose the main conditions of the respiratory system, such as airway obstruction caused by a foreign body, tracheal collapse, and stenosis. This is in addition to inflammatory or infectious processes, aspiration of secretions for cytological or culture tests and antibiograms. It is a complementary examination of direct inspection of the organ that allows the identification of changes and associate them with clinical findings^8^^,^ 9.

Histopathological examination allows microscopic analysis of the tissues to detect existing lesions, in addition to classifying them according to the extension, evolution, and nature of the local injury. The normal histological structure of the trachea is formed by different layers that are responsible for certain functions. The mucosa is lined by a pseudostratified ciliated epithelium composed of goblet cells that are responsible for the production and transport of secretions. The submucosa is composed of loose connective tissue that is rich in collagen and elastic fibers. The musclecartilaginous region is formed by cartilaginous rings, fibroelastic tissue, and the tracheal muscles. The rings have a C-shape and are composed of hyaline cartilage^10^^-^ 12.

The objective of this study was to evaluate the applicability of the sternocephalicus muscle flap in the repair of a partial tracheal defect with a new advancement technique and combined suture patterns.

## Methods

The project was carried out in the vivarium of the Postgraduate Course in Veterinary Surgery at the Faculty of Agricultural and Veterinary Sciences, Universidade Estadual Paulista “Júlio de Mesquita Filho” (UNESP), Campus Jaboticabal, Jaboticabal, SP, Brazil. The study followed the rules and ethical principles of animal experimentation approved by the National Council for Animal Control and Experimentation (CONCEA) and the institutional Ethics Committee on Animal Use. This project was filed under No. 006161/19.

Sixteen rabbits (*Oryctolagus cuniculus*; New Zealand White, healthy, male, weighing 3–3.5 kg) from the Central Animal Facility of the Faculty of Veterinary Medicine and Animal Science, UNESP, Campus Botucatu, Botucatu (SP), Brazil, were used as an experimental model. The animals were divided into four groups according to the period of postoperative evaluation. The groups were named as G7 (group of seven days), G15 (group of 15 days), G30 (group of 30 days), and G60 (group of 60 days), with four animals at each time point.

### Anesthetic protocol and surgical procedure

Anesthesia was initiated with pre-anesthetic medication consisting of chlorpromazine hydrochloride (0.5 mg/kg), and morphine (0.5 mg/kg). These were injected intramuscularly. Anesthetic induction was performed with intravenous propofol (10 mg/kg) and orotracheal intubation with an endotracheal tube (n = 3). Anesthesia was maintained by inhalation with isoflurane diluted in 100% oxygen through an anesthesia machine with a total flow of 1 L/min.

For surgical preparation of the animal, extensive trichotomy was performed from the ventral cervical region to the manubrium, antisepsis with 70% alcohol, and 2% chlorhexidine detergent. The surgical technique was performed following all aseptic principles, as well as the use of surgical material, gloves, gauze, swabs, and sterilized field cloths. The surgical team was the same for all the procedures.

**Figure 1:**
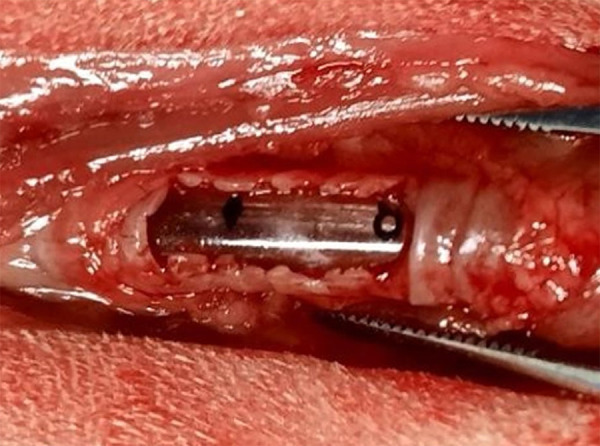
Photographic image of the surgical procedure for creating a partial defect in the trachea of a rabbit (*Oryctolagus cuniculus*), by resection of the fourth to eighth tracheal ring. Jaboticabal, SP, Brazil, 2021.

Source: elaborated by the authors.

The skin was incised from the midline of the ventral cervical region, caudal to the cricoid cartilage and extending to the manubrium. The subcutaneous tissue was divulged, followed by the location and removal of the sternohyoid muscle until the trachea was visualized and exposed in a delicate manner to avoid damage adjacent to the irrigation and local innervation. A defect was created in the ventral region of the trachea by resection of the fourth to eighth tracheal ring under the annular ligaments, with the aid of a #15 scalpel blade. This enabled the generation of a ventral partial defect, but in full thickness, exposing the tracheal lumen (Fig. 1).

**Figure 2:**
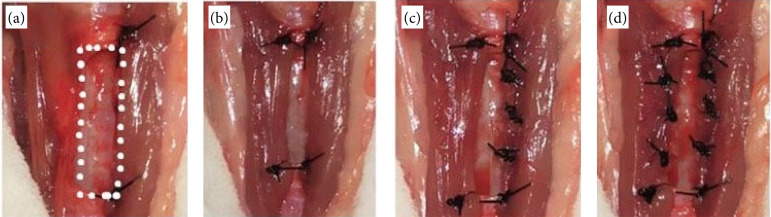
Surgical procedure for creating a partial defect in a rabbit’s trachea. **(a)** Partial tracheal defect in a rabbit (white dotted). **(b)** Bilateral advancement flap of the sternocephalicus muscle (white arrow) fixed by repair stitches at the end of the defect. **(c)** Border of the defect with suture of the simple pattern separated next to the musculature. **(d)** Fixation of each muscle belly anchored to the tracheal defect. Jaboticabal, SP, Brazil, 2021.

Source: elaborated by the authors.

**Figure 3:**
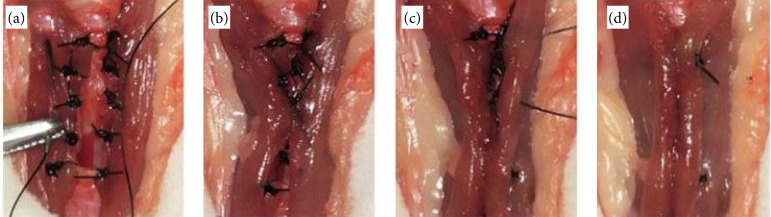
Surgical procedure for creating a partial defect in the trachea of rabbit. **(a)** Gelly-type suture between the musculatures. **(b)** Approximation and sealing of the tracheal defect. **(c)** Cranial and caudal end of the muscles sutured in the Wolf-type pattern. **(d)** Complete repair of the defect by bilateral advancement flap of the sternocephalicus muscles.

Source: elaborated by the authors.

The sternocephalicus muscles were identified, and the previously prepared defect was covered by them as a bilateral advancement flap. This was initially fixed using two sutures in a separate simple pattern distributed at each end of the defect. On each lateral border of the defect, three sutures were placed in a separate simple pattern along the musculature, and subsequent fixation, using 4-0 polypropylene (Fig. 2). The central region was positioned using a Gelly type suture, resulting in approximation, and sealing of the defect through the musculature. The cranial and caudal extremities of the muscles over the defect were positioned using wolf sutures (Fig. 3). After the completion of the technique, the subcutaneous tissue and the skin were approximated in a conventional manner.

### Postoperative evaluation

In the immediate postoperative period, hydrocortisone (50 mg/kg/IV) was used as a therapeutic protocol (single application). Also, tramadol hydrochloride (4 mg/kg/IM) and enrofloxacin (5 mg/kg/SC) were used. The animals were monitored and warmed until they regained consciousness and showed a normal obedience pattern. The same postoperative treatment with antimicrobials and anxiety was maintained for five days, with an interval of 12 h. The rabbits remained in individual cages for the management of the species, measuring 50 × 80 × 60 cm (height, length, and depth). They were provided with water, and commercial feed *ad libitum* in an air-conditioned environment throughout the period of analysis.

The rabbits were clinically evaluated periodically. Vital parameters such as breathing pattern, respiratory noises, presence, or absence of contamination in the surgical wound, subcutaneous emphysema, and animal behavior were monitored during the period.

The groups were euthanized seven, 15, 30, and 60 days after the surgical procedure. The euthanasia method complied with the ethical principles in animal experimentation established by CONCEA. It was carried out through the intravenous administration of propofol (dose-effect). After confirming the absence of corneal and pupillary reflexes, 20 mL of potassium chloride was administered intravenously to induce asystole.

### Tracheoscopy

Tracheoscopy was performed immediately after euthanasia. The device used was a 4 mm, 30° rigid endoscope (Karl Storzendoskope, Telecam SL NTSC micro-camera, Hopkins II optics with 250 Twin and Xenon 100 halogen cold light source). The endoscope was introduced into the oral cavity of the animal after gentle lateral traction of the tongue, progressing through the oropharyngeal region, until it was possible to visualize and film the larynx and trachea. Images were captured and recorded using an Acer Aspire 5741-5775 notebook.

The tracheal lumen was evaluated after euthanizing each animal in the appropriate groups and evaluation times. They were analyzed for the presence of secretion, exuberant granulation tissue, intraluminal sutures, and tracheal stenosis. Stenosis was determined according to the degree of lumen narrowing as follows: 0%, absent; < 25%, mild; 26–50%: moderate; and > 50%, severe. The presence of intraluminal suture stitches, secretion, and exuberant granulation were categorized as present or absent.

### Histopathological evaluation

After euthanasia, tracheas were dissected and removed up to 2 cm from the carina for histopathological analysis. The fragments were stored in flasks containing 10% formalin for at least 72 h and sent for slide preparation. Tracheal segments fixed in formalin were dehydrated in increasing series of alcohol, diaphanized, and embedded in histological paraffin, followed by semi-serial sections on a microtome (Leica model RM2145) with a thickness of 5 μm. Slides stained with hematoxylin and eosin (HE) were prepared for imaging under conventional light microscopy. Subsequently, the images were analyzed for regeneration and epithelialization of the tracheal mucosa at the injury site.

### Statistical analysis

Statistical tests were performed using the R Statistical software (R Project for Statistical Computing, version 4.2.0). The variables of the clinical, tracheoscopic, and histopathological evaluation parameters were subjected to descriptive analysis by the distribution of variables, Spearman correlation test, and Kruskal-Wallis’ comparison test. For the comparison test, the significance level was set at 5%. Therefore, all tests that obtained a p-value lower than 5% were considered significant.

## Results

Most of the operated rabbits had good surgical recovery without presenting serious alterations in the site of the procedure, such as suture dehiscence, subcutaneous emphysema, seroma, or contamination and secretion of the surgical wound. The most common clinical signs were tracheal stridor, present in the same amount in the 15- and 30-days groups (50%), and cough, predominantly in the 15-days group (50%). This did not interfere with the quality and survival of the animals, which remained active without changes in the respiratory system. The data distribution is presented in Table 1.

**Table 1:** Clinical, tracheoscopic and histopathological evaluation of the respective groups at seven, 15, 30 and 60 days, after flapping the sternocephalicus muscle in the repair of a partial defect in the trachea in a rabbit (*Oryctolagus cuniculus*).

Clinical evaluation						Tracheoscopy		Stenosis	Histopathological
Animal	Group (days)	Tracheal Stridor	Cough	Dyspnoea		Secretion	Lush Granulation		Epithelialization of the tracheal mucous
A1	7	+	-	-		+	+	-	-
A2	7	-	-	-		-	+	-	-
A3	7	-	-	-		-	+	-	-
A4	7	-	-	-		-	+	-	-

A1	15	+	+	-		+	-	-	-
A2	15	-	-	-		-	+	discreet	-
A3	15	-	-	-		+	-	-	-
A4	15	+	+	-		+	+	-	-

A1	30	-	-	-		-	-	-	+
A2	30	+	-	-		+	+	moderate	+
A3	30	-	-	-		-	-	-	+
A4	30	+	+	-		+	+	moderate	-

A1	60	-	-	-		-	-	-	+
A2	60	-	+	-		+	-	-	+
A3	60	-	-	-		-	-	-	+
A4	60	-	-	+		*	*	*	*

* Animal death;

+ gift;

absent. Source: elaborated by the authors.

Among the 16 operated rabbits, only one of the G60 died (6%) 12 days after the surgical procedure. This animal presented severe dyspnea, cyanosis, an orthopedic position, and rapid evolution until death. After post-mortem macroscopic evaluation, a large amount of serous secretion was noted, but without a local volume increase, suture dehiscence, or stenosis in the tracheal lumen.

**Figure 4:**
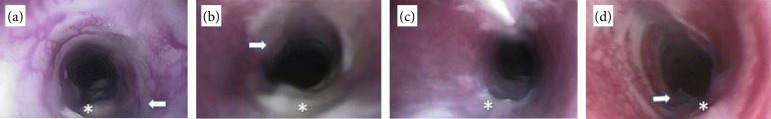
Representative tracheoscopic images of rabbits submitted to tracheal repair with muscle flap. **(a)** Animal from the 7-day group, presence of exuberant granulation over the muscular flap (*) and visualization of the suture thread (arrow). **(b)** Animal from the 15-day group, fibrin deposition on the flap and slight presence of secretion (*). Note mild stenosis due to intraluminal involvement (arrow). **(c)** Animal from the group of 30 days, showing good healing over the flap and no intraluminal involvement (*). **(d)** Animal from the 60-day group, showing good healing on the flap (*) and visualization of the suture thread (arrow). Jaboticabal, SP, Brazil, 2021.

Source: elaborated by the authors.

The main alterations observed by tracheoscopy were secretion in the tracheal lumen, exuberant granulation over the flap, and local stenosis (Fig. 4). The visualization of intraluminal suture points has been identified in different ways. However, there were no post-surgical complications as none of the rabbits had flap dehiscence or complications adjacent to the cervical region, including secretion or subcutaneous emphysema.

Intraluminal secretion was observed in animals in all groups, particularly in the 15-day group (75%). It has a mucoid aspect, with varied distribution and quantity on the tracheal wall and muscle flap. Exuberant granulation formation in the musculature was prevalent in the 7-day group (100%). As a phase of the healing process, examination showed fibrin deposition at the flap site. Regarding intraluminal structural impairment of the trachea, two (50%) animals in the 30-day group had a moderate degree of stenosis (Table 1). There was no severe respiratory compromise in animals that presented with tracheal stenosis.

The interface area between the trachea and the flap was evidenced by histological staining with hematoxylin-eosin (HE) and imaging using conventional light microscopy. It was possible to demonstrate the formation of a new epithelium in the region of the tracheal defect in the animals after 30 (75%) and 60 days (100%), completely. This covered the lumen in the region of the muscle flap (Table 1).

**Figure 5:**
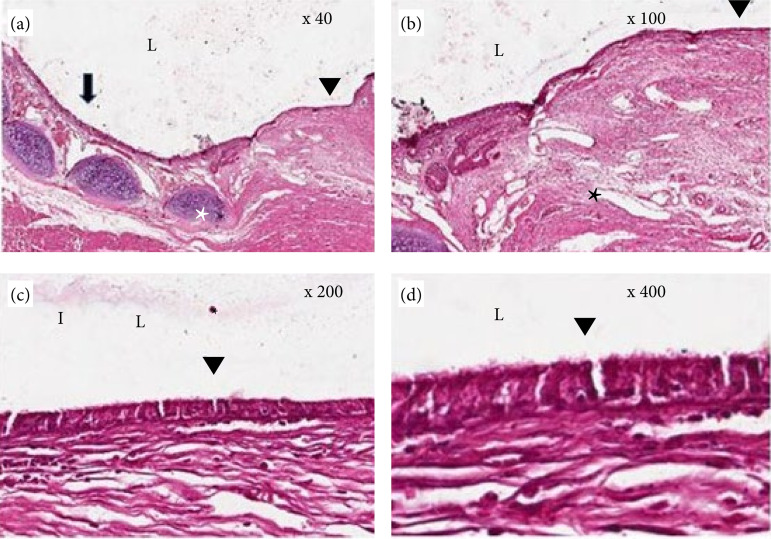
Hematoxylin-eosin color photomicrographs of the interface region between the last tracheal ring and the flap of the sternocephalicus muscle of rabbits represented the repair of the defect after 30 days. **(a)** Longitudinal section of the hyaline cartilage of the last tracheal ring (*). Normal respiratory mucosa (arrow). Ingrowth, typical of tracheal respiratory mucosa (arrowhead) over the flap region. **(b)** Longitudinal section of the defect transition region and flap implantation. Muscle fibers of the sternocephalicus muscle (*). **(c)** Area of inflammation located by discrete specific points of connective tissue formed by fibroblasts, presence of neovascularization and discrete focal and mononuclear inflammatory infiltrate. **(d)** Growth of new controlled pseudostratified epithelium, cilia, and goblet cells (arrowhead).

Source: elaborated by the authors.L: Lumen.

Complete respiratory mucosa was identified, which was newly formed by pseudostratified epithelium, goblet cells, and cilia. The area of injury was composed of discrete proliferations of immature connective tissue formed by reactive fibroblasts and focal and discrete mononuclear inflammatory infiltrates. In addition, there were areas of discrete neovascularization and passive hyperemia. There was no intense necrosis with a solution of continuity at the site. This showed good integration between the last tracheal ring and the stenocephalic muscle (Fig. 5).

As shown in Table 2, the Kruskal-Wallis’ test showed a significant p-value only for the variables of exuberant granulation (0.016*) and epithelization of the tracheal mucosa (0.002*). It can be said that there is a significant difference between the healing process of granulation tissue regression and regeneration by epithelialization with the increase in the number of the days of evaluation.

**Table 2:** Kruskal-Wallis’ comparison test, to identify the level of significance between the analyzed variable and the evaluation period in days (groups).

Variable	Days	Absent	Gift	p-value
Tracheal Stridor	7	3	1	0.483
	15	2	2	
	30	2	2	
	60	4	0	
Cough	7	4	0	0.617
	15	2	2	
	30	3	1	
	60	3	1	
Dyspnoea	7	4	0	0.179
	15	4	0	
	30	4	0	
	60	3	1	
Secretion	7	3	1	0.905
	15	1	3	
	30	2	2	
	60	2	1	
Lush granulation	7	0	4	0.016*
	15	2	2	
	30	2	2	
	60	3	0	
Stenosis	7	4	0	0.602
	15	3	1	
	30	2	2	
	60	3	0	
Epithelialization	7	4	0	0.002*
	15	4	0	
	30	1	3	
	60	0	3	

Source: elaborated by the authors.

**Figure 6:**
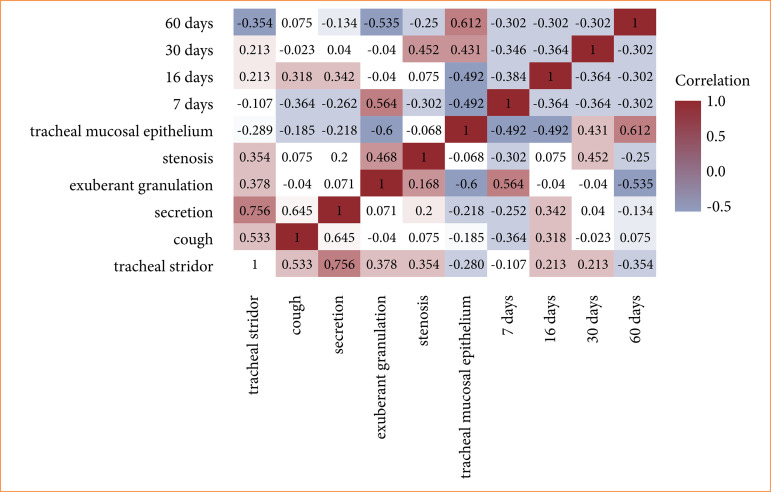
Spearman correlation analysis between the variables of tracheal stridor, cough, secretion, exuberant granulation, stenosis, and epithelialization of the tracheal mucosa, and their respective groups of seven, 15, 30, and 60 days.

Source: elaborated by the authors.

The correlation between the variables of different evaluations, using the Spearman test, showed that the tracheal mucosa epithelialization variable presented a reasonable negative correlation between exuberant granulation at seven and 15 days (Fig. 6). That is, when exuberant granulation is present, epithelialization of the tracheal mucosa tends to be absent. However, epithelialization showed a reasonably positive correlation at 30 days and a strong positive correlation at 60 days. Epithelialization of tracheal mucosa tends to occur over time. When other variables were concerned, the correlations were either weak or null.

The stenosis variable showed a reasonable positive correlation with tracheal stridor and exuberant granulation within the 30-day period. Thus, stenosis tends to occur when these variables are present. It showed a reasonable negative correlation at seven days; that is, during this evaluation period, the stenosis variable tends to be absent. Regarding other variables, the correlations were either weak or null (Fig. 6).

Secretion presented a reasonable positive correlation with tracheal stridor and a strong positive correlation with cough in the 15-day period. That is, when these variables are present, secretion tends to be present. However, there was a reasonable negative correlation at day 7 with cough. Therefore, no cough was witnessed during the initial evaluation period. For the other variables, the correlation was either null or weak (Fig. 6).

## Discussion

The main functions of the sternocephalicus muscle are the lateralization and flexion of the head and neck. Lateralization occurs through unilateral contraction and flexion through bilateral contraction of the muscles. It can also help in opening the mouth in species of mandibular origin^12^. After the surgical procedure, the rabbits were evaluated for movement in the cage and apprehension of food and water intake. All these parameters were normal. Therefore, the use of the sternocephalicus muscle by advancing and bilaterally suturing the bellies proved to be efficient in not compromising the movement of the head and neck.

Cough is an important physiological function of the respiratory tract, and its main actions are the removal and expulsion of unwanted or harmful particles, such as foreign bodies, cellular debris, and mucus^13^. Of the animals that showed clinical signs of cough, all had mucoid secretion in variable amounts, identified by tracheoscopy examination. This suggests an attempt to eliminate it and preserve the airway. Stridor is characterized by the presence of synchronous breathing noises due to airway obstruction that compromise the normal flow of air. Obstructive processes include the presence of mucus, foreign bodies, collapse, and tracheal stenosis^2^. The animals identified with moderate tracheal stenosis showed tracheal stridor in the clinical evaluation, which is consistent with the obstructive aspects that interfere with the patency of normal airflow.

Tracheoscopy allows direct visual inspection of the trachea, evaluation of tracheal collapse, luminal stenosis, and inflammatory and infectious processes. These were evident in the presence of secretion and mucosal tissue damage, in addition to neoplastic involvement^8^^,^ 14. After injury, tissue repair begins by stimulating the local cells in the injured region. The production of fibrin, in addition to promoting adherence to the wound, stimulates the migration of defense cells such as leukocytes, macrophages, neutrophils, and fibroblasts, which are important in local collagen deposition^15^. The healing process of maturation in the injured area leads to contraction of the tissue edges, which causes retraction and consequent intraluminal decrease in hollow organs^16^. Such information justifies the exuberant granulation and stenoses identified by tracheoscopic examination and is compatible with the phases and physiological processes of healing.

Repair of the tracheal defect is an important characteristic of the preservation of the biomechanics of the organ and maintenance of air flow by the different pressures exerted during breathing. The use of homologous muscle grafts or tridimensional fabrication prostheses proved to be efficient at the implanted site, supporting intratracheal pressure and preventing organ collapse^16^^,^ 17. Through tracheoscopy, it was possible to observe a low rate of tracheal stenosis in the operated animals, and, even when present, they did not show severe impairment of respiratory function or survival in the evaluated time. This corroborates previous studies on the maintenance of biomechanical properties.

Recent research has highlighted the use of prostheses, printed in three dimensions with biocompatible material, in the reconstruction of tracheal defects. These allow biological integration and biomechanical function compatible with the organ. Jung et al.^17^ evaluated the reconstruction of a partial defect in the trachea using a three-dimensional polyurethane prosthesis and found the formation of a fully functional respiratory mucosa with cuboidal epithelium, cilia, and goblet cells on the prosthesis after 60 days of implantation^17^. In the present study, it was possible to demonstrate the formation of a new respiratory mucosa on the muscular flap after 30 days of evaluation. It is assumed that the conditions that imply the healing process from the sternocephalicus muscle cause a faster local regenerative process than the use of prostheses.

Local revascularization is necessary for tracheal tissue regeneration. The studies carried out by Delaere and Raemdonck^18^ showed that, by wrapping the trachea over a well-vascularized muscle fascia flap, it allowed significant revascularization that was achieved by rapid growth from capillary buds of the remaining recipient vessels, united to the donor vessels of the muscular segment. Thus, direct vascular anastomoses occur between the tissue flap and the adventitia of the trachea.

Homologous muscle grafts have been also used for tracheal repair in dogs with good results. However, in these cases, histological analysis showed progressive replacement of the muscle fibers of the graft by mature fibrous connective tissue and less local vascularization. This is probably because it was a free segment with no proper irrigation^16^. It is estimated that the presence of proper irrigation to the muscular flap implies better conditions in the local healing and regenerative processes. This is also justified by the presence of intact muscle fibers and discreet proliferation of connective tissue, in addition to greater vascularization, as seen in the histological section.

The airway mucosa has a high proliferative capacity, which renews itself continuously after superficial injuries, and its regeneration occurs above the basement membrane. This continues if the stem cells are not compromised. However, in severe injuries with damage to the submucosa, tissue repair occurs through collagen deposition and healing^18^. However, further analyses should be carried out to investigate the regenerative process from the migration of epithelial basal cells from the wound margins in contact with the implanted tissue ends in the epithelialization process.

## Conclusion

The repair of a partial tracheal defect in a rabbit, as an experimental model, using the sternocephalicus muscle is satisfactory because of the anatomical availability of the muscle, its own vascular support, and structural maintenance, without compromising or serious alterations to the respiratory system. In addition, it allows epithelial regeneration from the implanted tissue bed, a process that is important for physiological maintenance of the respiratory mucosa.

## Data Availability

All data sets were generated or analyzed in the current study.
